# Patient and health system level barriers to and facilitators for tuberculosis treatment initiation in Uganda: a qualitative study

**DOI:** 10.1186/s12913-022-08213-w

**Published:** 2022-06-28

**Authors:** Stella Zawedde-Muyanja, Yukari C. Manabe, Adithya Cattamanchi, Barbara Castelnuovo, Achilles Katamba

**Affiliations:** 1 The Infectious Diseases Institute, College of Health Sciences, Makerere University Kampala, Mulago Hospital Complex, P.O. Box 22418, Kampala, Uganda; 2 Division of Infectious Diseases, Department of Medicine, Johns Hopkins University School of Medicine, Baltimore, MD USA; 3 Division of Pulmonary and Critical Care Medicine and Center for Tuberculosis, University of California San Francisco, San Francisco California, USA; 4 Uganda Tuberculosis Implementation Research Consortium, Kampala, Uganda; 5 Clinical Epidemiology and Biostatistics Unit, Department of Medicine, Makerere University College of Health Sciences, Mulago Hospital Complex, P.O. Box 7072, Kampala, Uganda

**Keywords:** Tuberculosis, Treatment initiation, Patient, Health systems, Barriers, Facilitators, Qualitative, Uganda

## Abstract

**Background:**

The WHO END TB strategy targets to place at least 90% of all patients diagnosed with Tuberculosis (TB) on appropriate treatment. In Uganda, approximately 20% of patients diagnosed with TB are not initiated on TB treatment. We sought to identify the patient and health system level barriers to and facilitators for TB treatment initiation in Uganda.

**Methods:**

We conducted the study at ten public health facilities (three primary care, four district and three tertiary referral hospitals). We carried out in-depth interviews with patients diagnosed with TB and key informant interviews with health managers. In addition, we held focus group discussions with healthcare workers involved in TB care. Data collection and thematic analysis of transcripts was informed by the Capability, Opportunity, Motivation and Behavior (COM-B) model. We identified relevant intervention functions using the Behavior Change Wheel.

**Results:**

We interviewed 79 respondents (31 patients, 10 health managers and 38 healthcare workers). Common barriers at the health facility level included; lack of knowledge about the proportion of patients not initiated on TB treatment (psychological capability); difficulty accessing sputum results from the laboratory as well as difficulty tracing patients due to inadequate recording of patient addresses (physical opportunity). At the patient level, notable barriers included long turnaround time for sputum results and lack of transport funds to return to health facilities (physical opportunity); limited TB knowledge (psychological capability) and stigma (social opportunity). The most important facilitators identified were quick access to sputum test results either on the date of first visit (same-day diagnosis) or on the date of first return and availability of TB treatment (physical opportunity). We identified education, restructuring of the service environment to improve sputum results turnaround time and enablement to improve communication of test results as relevant intervention functions to alleviate these barriers to and enhance facilitators for TB treatment initiation.

**Conclusion:**

We found that barriers to treatment initiation existed at both the patient and health facility-level across all levels of the (Capability, Opportunity and Motivation) model. The intervention functions identified here should be tested for feasibility.

**Supplementary Information:**

The online version contains supplementary material available at 10.1186/s12913-022-08213-w.

## Introduction

During the 2018 United Nations high level meeting on Tuberculosis (TB), countries committed to treat 40 million people with Tuberculosis by 2022 [1]. This commitment corresponds to the End TB strategy’s target to improve TB treatment coverage (the proportion of incident TB cases notified in a given year) to 90% by 2025 [2]. In 2019, 7.1 million new cases of TB were notified globally to the World Health Organization (WHO) against an estimated 10 million cases representing 70% treatment coverage [3] This gap between estimated incident TB cases and those notified to the WHO is due to a combination of under-diagnosis and under-reporting. Under-diagnosis results from people not accessing TB services or not being tested for TB when they do. In sub-Saharan Africa, data from 13 national TB prevalence surveys showed that one third of all participants with TB symptoms had sought care for these symptoms but had not been tested for TB. Of these, 75% had sought care at public health facilities [4]. On the other hand, under-reporting results from people either accessing TB treatment outside the public health system or being diagnosed with TB within the public health system but not being initiated on TB treatment (pretreatment loss to follow-up). In sub Saharan Africa, only about 10% of TB patients receive treatment outside the public healthcare system [4, 5] Pretreatment LFU therefore accounts for the majority of underreporting with up to 38% of patients who are diagnosed with TB not initiating TB treatment [6].

Uganda is one the 30 high TBHIV burden countries in the world [7]. The 2015 prevalence survey showed that the country’s TB prevalence, at 253/100,000 [5], was much higher than had been previously estimated(159/100,000) [8]. Following this survey, the Uganda National TB and Leprosy program (NTLP) implemented various interventions to improve TB treatment coverage including training of healthcare workers and increasing access to novel diagnostic tests like GeneXpert testing. These efforts resulted in an increase in the number of TB cases started on TB treatment from 45,000 (50% of estimated incident cases) in 2016 to 60,000 (68% of estimated incident cases) in 2020 [9]. However, the proportion of estimated incident cases started on TB treatment remains below the 90% target set by the WHO END TB Strategy. Pre-treatment LFU (the loss of patients between TB diagnosis and treatment initiation) is one of the persistent causes of suboptimal TB treatment coverage which has not significantly improved despite improvements in TB diagnostic modalities. Previous work from Uganda has documented pretreatment LFU rates of up to 30% of all diagnosed TB patients annually [10–14]. This work has shown that tertiary health facilities with high patient volumes have the highest rates of pretreatment LFU (10, 12) and that at these health facilities, losses are highest among children < 15 years with up to 45% of them not being initiated on TB treatment [14]. To further improve TB treatment coverage, the NTLP will need to, in addition to other interventions, improve treatment initiation among patients diagnosed with TB.

Behavioral theory represents a systematic way of understanding events or situations [15]. In public health practice, behavioral theories provide an understanding of the processes underlying the implementation of evidence-based interventions and provide a framework to enable the design of more effective implementation strategies [16]. The Capacity, Opportunity, Motivation and Behaviour (COM-B) model is a behavioral theory that captures a range of mechanisms involved in behavior change and links them to a comprehensive structure of interventions and policy functions that can be employed to influence behavior [17]. At its core is a behavior system (the COM-B) that states that for an individual to engage in a given behavior (B) at a given moment they must have i) physical and psychological capacity (C); ii) social and physical opportunity (O) and, iii) the desire to do this activity more than other competing activities (M). Connected to this model is an implementation framework, the Behavior Change Wheel [17] (BCW). The BCW contains nine intervention functions tailored to address specific identified barriers e.g. education can be used to increase the psychological capability of engaging in a behavior by providing information about its health consequences while environmental restructuring can be used to add objects to the environment e.g. prompts and cues that increase the physical opportunity of engaging in a behavior.

In this qualitative study, we applied the COM-B model to identify the barriers to and facilitators for TB treatment initiation at public health facilities in Uganda. We then used the intervention functions outlined in the BCW to identify interventions that can overcome these barriers with the aim of increasing the proportion of patients initiated on TB treatment at these health facilities.

## Methods

### Study setting

The study was conducted in ten public health facilities in central Uganda from March to June 2019. The health facilities were chosen to represent the different levels of the healthcare system and included (three primary care, four district and three tertiary referral hospitals). All health facilities provide TB diagnostic and treatment services. Patients are screened using a five-question symptom screen at several care delivery points within the health facilities including the outpatient clinics (OPDs), HIV clinics, nutrition clinics and inpatient wards. Patients with presumptive TB are then asked to submit sputum samples which are sent to the laboratory for GeneXpert testing. The laboratory staff convey the results of sputum testing to the requesting clinic staff who in turn convey these results to the patients. Patients diagnosed with TB are then initiated on TB treatment at the TB clinics. TB treatment is offered on an outpatient basis unless there is another indication for inpatient care. All health facilities use standardized national paper-based registers to record patients with signs and symptoms of TB (TB presumptive registers); patients with a bacteriological confirmation of TB (TB laboratory registers); and patients started on TB treatment (TB treatment registers). Patients who do not initiate TB treatment are traced by community healthcare workers using patient locators (phone numbers and physical addresses) provided in the presumptive TB registers.

### Study participants

To elicit patient-level barriers to and facilitators for TB treatment initiation, we carried out in-depth interviews (IDIs) with two purposively selected groups of respondents a) patients who successfully initiated TB treatment within 2 weeks of diagnosis and b) patients who did not initiate treatment within 2 weeks of diagnosis i.e. those who experienced pre-treatment LFU.

To obtain a wide range of perspectives, we used a maximum variation sampling strategy. We included respondents who were HIV+ and those who were HIV-; respondents who were younger (less than 25 years) and older (more than 60 years); and those living less than 5 km from the health facility and more than 25 km from the health facility. In each category, we included both male and female respondents.

To elicit health facility-level barriers to and facilitators for TB treatment initiation, we carried out focus group discussions with health workers providing TB services. To get representative views, we held one focus group discussion (FGD) at each level of the healthcare system (primary health facility, district hospital and tertiary referral hospital). We purposively selected health facilities which initiated < 80% of all diagnosed patients on TB treatment. In addition, we held one FGD at a health facility which initiated > 90% of all diagnosed patients on TB treatment. This health facility was added to improve our understanding of health facility level facilitators for TB treatment initiation.

In addition, we carried out key informant interviews with health managers. We selected one health manager from each hospital. Health managers were selected from those in charge of the laboratories, the outpatient clinics, the HIV clinics or the inpatient wards.

### Study instruments

#### Patient interview guides

Using the COM-B model, we developed an in-depth interview (IDI) guide for patients. Each patient interview guide included six to eight open-ended questions exploring barriers to and facilitators for TB treatment initiation such as: capacity to recognize TB symptoms; understanding of the diagnostic process and how to receive test results; ease of accessing TB diagnosis and sputum test results; ease of accessing TB treatment as well as attitudes towards TB and its treatment. The patient interview guides were drafted in English and refined during a pilot study carried out at a primary health facility which was not part of the study. They were then translated into the local languages (Luganda or Lusoga) spoken in the study area.

#### Healthcare worker interview guides

We developed key informant interview guides (for health managers) and focus group discussion guides (for healthcare workers). All health worker interview guides were based on the COM-B model and explored healthcare workers’ knowledge about the proportion of patients not initiated on TB treatment at their health facilities; aspects of daily workflow that influenced treatment initiation among patients diagnosed with TB; availability of monitoring systems to flag patients who were not initiated on TB treatment; availability of patient follow-up mechanisms to ensure that any patients flagged by the system are followed up and initiated on TB treatment and healthcare workers’ attitude towards their role in ensuring that patients diagnosed with TB are started on TB treatment. Finally, questions about which intervention functions could be implemented to address elicited barriers to and enhance facilitators for linkage to TB treatment were included. Interview guides were drafted in English and piloted at a primary health facility which was not part of the study.

### Data collection procedures

#### Patient interviews

Patients successfully initiated on TB treatment were selected from those attending clinic refill visits and invited to participate in the study while those who experienced pre-treatment LFU were selected from those successfully traced by the study team. Patients who declined to participate in the study were replaced by other patients who fit the selection criteria. All patients were interviewed only once at the health facility by either the study investigator (SZM), a female medical doctor with experience in TB care provision or her assistant (JN), a male study nurse. Both SZM and JN had additional training in research methods and qualitative research. The study team was assisted by health facility personnel who were fluent in the local language used. At the beginning of each interview, the researchers introduced themselves and explained their role in and reasons for conducting the research study. Permission was sought from the patients to take notes and audio record the interviews. All patient interviews were conducted in the local languages and lasted 20-30 minutes. Interviews were carried out until saturation was achieved.

#### Healthcare worker interviews

Healthcare workers were purposively selected from those involved in any of the following TB care activities (health education, screening, diagnosis, treatment and community follow-up). Selected healthcare workers were approached by the study investigators (SZM and JN) who explained their role in and reasons for conducting the research study and requested the healthcare workers to take part in the study. FGDs were held at the respective health facilities at the end of the workday and were led by SZM while JN took field notes and audio recorded the discussions. All FGDs were conducted in English and lasted 30-60 minutes. Discussion were held until saturation was achieved.

Health managers were interviewed by the study nurse (JN) at their respective posts after making appointments with them over the phone. Interviews were audio recorded and field notes were taken. All interviews held once, were conducted in English and lasted 30-60 minutes.

### Data analysis

At the end of the interviews, audio recordings from the patient interviews were transcribed and translated by research assistants proficient in the local language used and English. Healthcare worker interviews were transcribed by the study nurse. The anonymized transcripts were analyzed and coded by the study investigator (SZM) and another independent qualitative researcher who was not one of the study investigators. We used a deductive approach applying the COM-B model as the framework for analysis. We resolved discrepancies by mutual agreement to produce an analysis of the barriers to and facilitators for TB treatment initiation. Finally, using the BCW framework, we identified intervention functions that could alleviate the barriers to and promote facilitators for TB treatment initiation. Data analysis was carried out using NVivo software.

#### Ethics statement

This study was approved by the Makerere University School of Medicine Research and Ethics Committee of the College of Health Sciences (Ref: 2016-132) and by the Uganda National Council of Science and Technology. Before each interview, participants provided written informed consent including consent to audio-record the interviews. For participants below the age of 16 years, written informed consent was obtained from a parent or legal guardian while the participant provided written assent to participate in the study. All methods were performed in accordance with the good clinical practice and other relevant guidelines and regulations.

## Results

### Demographic characteristics of study participants

We interviewed 31 patients- 15 who had been successfully initiated on TB treatment 16 who had not. In addition, we held FGDs with 38 healthcare workers from different categories (Table 1). Finally, we conducted 10 KIIs with health managers (five heads of clinic and five laboratory managers - two of whom also double as district laboratory focal persons).

**Table 1:** Demographics characteristics of study respondents for in-depth interviews and focus group discussions

Characteristic	N (%)
**In-depth Interviews (*N*** **= 31)**	
Successfully initiated on TB Rx	15 (48)
Not successfully initiated on TB Rx	16 (52)
**Sex**	
Male	21(68)
**Age**	
15-24	9 (29)
25-34	10 (32)
35-44	7 (23)
> 45	5 (16)
**Health facility level**	
HC IV	3 (10)
District hospital	12(38)
Tertiary referral hospital	16 (52)
**HIV status)**	
HIV-negative	18 (58)
HIV-positive	13 (42)
**Focus Group Discussions (*N*** **= 38)**	
**Cadre**	
Clinical Officers	9 (24)
Laboratory Officers	10 (26)
Nurses	14 (37)
Community Healthcare workers	5 (13)
**Sex**	
Male	22 (58)
**Years at current post**	
< 2 years	6 (16)
2-5 years	15 (39)
6-9 years	13(34)
≥ 10 years	4 (11)

### Barriers to TB treatment initiation

For patients to get initiated on TB treatment, they must first a) recognise TB symptoms and seek care at an appropriate healthcare facility, b) submit sputum samples for analysis c) receive the sputum analysis results and d) get started on TB treatment. We present below, the themes that emerged as barriers and facilitators along this care cascade at both the health system and patient level. These themes are also represented according to the COM-B domains in Table 2.

**Table 2:** Barriers to and Facilitators for TB treatment initiation among patients diagnosed with TB at public hospitals in Uganda

Domain	BARRIERS	
	Health Facility Level	Patient-level
**Capability**		
Psychological	Lack of awareness of the magnitude of pre-treatment LFU at the health facilities	Lack of TB knowledge among patients
**Opportunity**		
Physical	Insufficient time and space for patient education	Long turnaround time for sputum results
	Late delivery of sputum samples to the laboratory due to batched sample collection.	
	Lack of tools to monitor TB treatment initiation among patients diagnosed with TB	Lack of transport funds to retrieve sputum results
	Difficulty retrieving sputum test results by most clinics due to lack of collaboration with the laboratory	Lack of time to retrieve sputum results.
	Difficulty tracing patients due to inadequate recording of patient locators (physical addresses or phone numbers).	Conflicting messages from healthcare workers about sputum results retrieval.
Social	Disinterest in performing sputum analysis.	Stigma
**Motivation**		
Automatic	Staff discomfort with performing sputum analysis	TB associated stigma
Reflective	Reduced motivation to collect sputum results due to difficulties accessing the laboratory.	Misconceptions about susceptibility to TB disease
**FACILITATORS**		
**Capability**		
Psychological	Knowledge of QI improvement methods	Prior knowledge of TB
**Opportunity**		
Physical	Availability of cough screeners to facilitate TB screening	Availability of sputum results on same-day as first clinic visit.
	Availability of community healthcare workers to trace patients in the community	Ease of access to TB treatment after results retrieval
	Ability to notify patients about their sputum test results through phone calls.	
Social	Health facility norms e.g. escorting patients diagnosed with TB to the TB clinics.	
**Motivation**		
Automatic		Desire to get well and provide for/take care of family
		Desire to protect family members from TB Courage obtained after psychological counselling
Reflective	Healthcare workers’ sense of duty to the patients and community	Trust in quality of care at larger public health facilities.

#### Barriers to submitting sputum samples for analysis

Several patients interviewed did not recognize signs and symptoms of TB. When these patients presented to care, healthcare workers did not adequately explain the possible cause of their symptoms or why they were being examined. As a result, patients left the health facility without a clear understanding of the disease. This may have affected their willingness to come back to the health facility to retrieve their sputum results or to initiate TB treatment. One patient recounted that the healthcare worker only told him to produce a sputum sample with no explanation for why the sputum sample was being collected.

*“When I reached him, the healthcare worker gave me a bottle and said, ‘go and spit in it, after you spit in it, bring it back to me.’ ‘ I know what I will do with it.’*” *[IDI_Patient].*

Healthcare workers acknowledged that patients were not adequately counselled before being tested for TB. In the outpatient clinics (OPDs), large patient volumes coupled with lack of private spaces for counselling patients made the provision of adequate patient education and counselling difficult.

*“OPD is totally different [from the HIV clinic] because when patients come, they are seated there. The person screening is seated in a crowd so that patients may not have enough time to ask each and everything or even reach the extent of counselling, actually counselling is also another gap. We are not giving our clients enough time especially TB patients.” [FGD _Nurse OPD].*

However, even in the HIV clinic where private spaces for counselling were availed, staff reported that human resource shortages sometimes compromised the quality of patient education.

*“The challenge is because of work load, you are here alone, you are filling this register, the other register, patients’ cards and you end up thinking. … may be this patient I have given enough talk, maybe she will come back, she will not get lost.” [IDI_Head Nurse HIV Clinic].*

To ensure that patients with presumptive TB submit sputum samples for TB testing, healthcare workers often asked them to produce sputum samples at the outpatient clinics (instead of referring the patients to the labs). Healthcare workers then delivered all the sputum samples to the laboratory in one batch, often at the end of the clinic day. Batched delivery of sputum samples, although designed to reduce patient losses between screening and sample collection/processing, inadvertently contributed to prolonging turnaround times for sputum test results.

*“For every patient who comes at OPD, we screen for TB. If that person is presumed to have TB, we enter that name into the presumptive TB register then we give that person a sputum container and tell them to go and cough and bring the sputum to me. We then put the sample in that cooler over there. After we have finished the clinic, we take the samples the lab.” [FGD _Nurse OPD].*

#### Barriers to receiving sputum analysis results

When we interviewed both patients and healthcare workers, receiving sputum test results emerged as the most significant barrier to TB treatment initiation. Both patients and healthcare workers reported that it took up to 48 – 72 hours (and occasionally as long as 5 days) to get sputum test results. These delays sometimes caused dissatisfaction among patients. One patient explained that she gave up on receiving her sputum test results after waiting in line all day and then being told to come back the next day.

*“I took there my sputum then I waited the whole day, they were calling other people, but they were not calling me until I walked up to the window and said, “musawo (healthcare worker) what about my results?”, he asked “which sample did you bring?” I said sputum. Then he told me “ah, no you come back tomorrow”. I did not come back. I went to look for another solution to my problem.” [IDI_Patient].*

Healthcare workers also expressed additional concerns that even when sputum test results were ready, it was difficult for the clinic staff to retrieve them because the laboratories, particularly the ones fitted with a biometric lock, were sometimes inaccessible. This reduced the nurses’ motivation to collect sputum results. One of the nurses said she had stopped going to the laboratory because the process was laborious, time-wasting and interfered with the rest of her workday.

*“Nowadays I have withdrawn. I have relaxed a bit. At times I would go picking results and the lab is locked. You know, now they use password, maybe password I don’t know. At times, it is not open, and you know when you are busy and you are kept outside standing, waiting, you are not given results …. So, I stopped going.” [FGD_Nurse OPD].*

To avoid long waits at the health facility, clinic staff often asked patients to return after 48-72 hours or if HIV infected to collect their sputum test results at the next clinic visit. Occasionally, clinic staff asked patients to wait at home until they received a phone call notifying them that their sputum results were ready. These mechanisms although intended to ease the process of receiving test results introduced additional barriers. Some patients could not find time off work or school engagements to come back to the health facility to retrieve sputum test results while others couldn’t afford a return journey to the health facility and stayed at home for considerable periods of time waiting to raise the transport fare.

*“When I took the sputum, the healthcare worker told me to come back after two days but I did not have anywhere to stay near the hospital, so I went back to the village. I delayed there because I did not have the transport [fare]. You know for us in the village, again transport to come back is difficult … ...I took two weeks before I came back for my results.” [IDI_Patient].*

Secondly, healthcare workers sometimes forgot to place phone calls to patients which resulted in patients staying at home for extended periods waiting for news of their test results. Two patients we interviewed stayed home for longer than a month. One of them, whose HIV clinic visits were 3 months apart waited 6 weeks to receive sputum test results.

*“I gave my sputum at the HIV clinic more than a month ago. The nurse told me to give my phone number, that she would call me if results came out. I went home and waited. I could not call the clinic because they did not give me their phone number, they took my number, but they did not give me their number. I have been planning to come back to the HIV clinic and ask about my results.” [IDI_Patient].*

Laboratory staff acknowledged the long turnaround time for sputum analysis and explained that this was due to the high volume of sputum samples that each laboratory had to process. They explained that in addition to processing sputum samples from within their hospitals, most laboratories supported GeneXpert testing for several primary health facilities within a 25 km radius.

*“We have a challenge with GeneXpert. It’s a four module. Every after two hours it releases four results, but you may find that, a day we receive between 30-40 samples. So, that means, if in a day, we can release like 12-16 results, the following day, we may have a balance of close to 25 or even 30. So, that means that patients will have to wait for like 48 hours or even more, so that becomes a challenge.” [KII_Lab Manager].*

Although clinic staff acknowledged that the laboratories had heavy workloads, they also noted that laboratory staff were disinterested in analysing sputum samples which further contributed to the long turnaround times.

*“Lab people, maybe I can say it’s their attitude. Some don’t want to work on the samples …… They have left that sputum work for some individuals in lab. Once those individuals are not present, you will first go there and beg … … ‘please, please we are not getting results.’ Until maybe you go to the person in-charge and say, ‘what is happening?’. Then he looks for someone who will run those samples.” [FGD _HCW OPD].*

*“I don’t know may be its others who don’t like doing that ‘dirty work’ of sputum analysis. Because even they can tell you frankly that, ‘not me, I can’t do that sputum [analysis].’ You ask why? They say ‘NO, those reasons are mine.’” [FGD_HCW OPD].*

Healthcare workers reported that the prolonged turnaround times for sputum test results were a significant health facility level barrier which interacted with other patient-level barriers e.g. distance from health facility and lack of transport fares to further decrease the likelihood of successfully initiating patients on TB treatment.

*“You don’t know what it means for a patient who comes may be from Buyende or Irapa [50 kms away] then you tell them.... ‘come back tomorrow for your results’. Then they come, no results. If they are patient enough, they go back but they have used transport [fares]. They come back on the third day, again no results. Such a patient will never come back.” [FGD_HCW OPD].*

#### Barriers to initiating patients on TB treatment

After receiving sputum test results from the laboratories, clinic staff communicated the results to the patients either physically when patients next visited the clinic or via the phone. Clinic staff concurred that it was easier and faster to communicate results to patients via the phone. However, sometimes they failed to do this because some clinic phones had no credit while other clinics did not have desk phones at all.

*“In the outpatient clinic (OPD) we sometimes call those ones whose results come later and they have given us [phone]contacts. However, we don’t have a telephone, we don’t have a telephone in OPD. So, when we get the results, we either move to TB clinic and or to ART clinic. But even sometimes you go, you find that the phones are being used by someone else then you have to keep moving [up and down] like this.” [FGD_Head Nurse OPD].*

At the clinics which had phones in place, staff explained that they were not always able to successfully communicate test results because the phone numbers recorded in the presumptive TB registers were inaccurate. Some patients could not accurately recall phone numbers particularly those belonging to family or community members while others were reluctant to receive phone calls that might contain TB related information due to stigma.

*“One of the challenges some patients are stigmatized, they think having TB is a sign of having HIV so because of that stigma some of them give wrong [phone]numbers. Someone gives you a [phone] number and when you call it, it’s off.” [KII_ Laboratory Manager].*

Patient confirmed the existence of TB associated stigma within their communities which made dealing with a TB diagnosis difficult. Some patients did not want to associate with a TB diagnosis because they thought it was a familial disease which did not exist within their families.

*“For me, mainly I knew that TB is a family disease. One is born with TB so for me, I could not think that I can get TB because those days you could go to the health facility for antenatal care and they ask you whether there is anyone with TB in your home. Then you would reply yes or no. In my family, I have never had anyone with TB so I couldn’t think about TB.” [IDI_Female patient].*

Others explained that a TB diagnosis made them feel less human and created a risk of humiliation and isolation from their families.

*“What was in my mind [when I was diagnosed with TB] was that they were going to isolate me and start to throw food to me like a dog … …*.” *[IDI_Patient].*

When patients could not be contacted by phone, community health workers physically traced them from within their communities. The biggest barriers to patient tracing were the lack of consistent funding and inability to trace patients due to incomplete locator information in the presumptive TB register.

“*For patient addresses, you have to know the sub county, the parish and the zone … …*. *I* *have traced patients, you find the patient has an address of Nambwiguru, Nambwiguru is a sub-county which has many parishes and zones so if you only record the address as Nambwiguru now which parish I am going to begin entering? and now if I enter a parish which zone will I begin asking? That is the challenge.” [FGD_Community healthcare worker].*

Clinic staff gave several reasons for incomplete recording of physical addresses including inattention by some healthcare workers, heavy workload and lack of space to record patients’ physical addresses on the available laboratory request forms.

*“But then you will also find that some other clinician in the room will write a requisition for GeneXpert using these normal forms which are not meant for GeneXpert and will not put all the information required.” [FGD_ Lab Technician].*

The final significant health facility level barrier to TB treatment initiation was the fact that clinic and lab staff were not aware that a significant proportion of patients diagnosed with TB were not initiated on TB treatment. Healthcare workers wrongly assumed that all patients diagnosed with TB eventually started on TB treatment. This misconception was partly because health facilities did not regularly reconcile their TB laboratory registers with their TB clinic register in order to systematically monitor linkage to TB treatment.

*“We don’t have any way of monitoring whether patients have started TB treatment, but I would say we just need to get one on board because for HIV we have, we report weekly those diagnosed with HIV and those linked to ART (antiretroviral therapy).” [KII_TB Focal Person].*

### Facilitators for TB linkage to treatment

We identified several key facilitators for TB treatment initiation. Patients who were successfully initiated on TB treatment reported prior knowledge of or experience with TB. Patients with prior TB knowledge (acquired from media campaigns or at community health talks) reported a better understanding of their TB symptoms.

*“Yes, I had heard that when you cough for like a week, it’s a sign of TB, so when I got cough about a week without getting better, I thought to myself that it might be TB so I went to the hospital. They tested me and told me that it was TB and started me on treatment.” [IDI-Patient].*

On the other hand, patients who had prior experience with TB through observing either a family or community member successfully complete TB treatment were motivated because they were convinced that early treatment led to a better treatment outcome and that it would protect their family members from getting the disease.

*“As I told you that chairman on the village cured. Though not all people on the village got to know, but us who knew about it we saw that he cured. When the health worker told me that I have TB, I got scared a bit but for the sake of protecting my young children at home, I later accepted, and I said, ‘I am ready to do it or getting drugs or what.’” [IDI_Patient].*

A short turnaround time for sputum results (results being available on the date of the initial clinic visit or of first return) combined with availability of TB medicines were strong facilitators of linkage to TB treatment. Many of the patients who were successfully linked to treatment reported receiving their sputum results with relative ease and starting treatment as soon they presented their sputum results to the TB clinic.

*“They told me to give them sputum, I took it to the lab for examination. They told me to come back after a day. I came back on the day they told me to come back and I got the results. I took them to the health worker who started me on treatment. I did not get any problem.” [IDI_Patient].*

Some health facilities instituted additional quality assurance measures to promote initiation on TB treatment for patients diagnosed with TB. One health facility laboratory only received sputum samples if accompanying laboratory forms were adequately filled so that patients could be traced back to the referring clinic while another physically escorted and handed over patients diagnosed with TB to the TB clinic where they were initiated on TB treatment.

*“But here in the lab before a sample enters in the main lab, you must cross check everything … … If you receive a sample, that has no, say presumptive TB number, you cannot go ahead and work on this sample. Because we have different points of care …… … now you see this one it has HIV clinic, it can easily be traced where the patient come from let’s say maybe from HIV clinic, general ward, maternity or from OPD.” [KII Lab Manager].*

Some health facilities called back patients for treatment initiation or trace them in the community if their sputum test results were positive for TB.

*“So, for the positive results we have to call them, they come and pick their results, they go to TB ward and they start on treatment. But they are some who do not have the [phone] contacts. We try to use the tracking tool from HIV clinic where we put the address so that we can track for that patient.” [KII_HIV Clinic Manager].*

Healthcare workers were also motivated to start patients diagnosed with TB on treatment out of a sense of duty to the community.

*“When we start patients on TB treatment, we are improving on infection control, we are reducing on the rate of spread of TB in the community … ..” [KII TB Focal Person].*

### Identifying potential intervention functions to address barriers to and enhance facilitators for TB treatment initiation

Together with stakeholders, we identified modifiable barriers and linked them to appropriate intervention functions using the Behaviour Change Wheel. For example, healthcare managers highlighted the importance of increasing awareness about the magnitude of pretreatment LFU among healthcare workers through education. Healthcare workers also noted that they needed to redesign their workflow so that sputum samples could get to the laboratory earlier in the day to reduce diagnostic delays and enable same day diagnosis (environmental restructuring). Further, healthcare workers in the outpatient clinic observed that the provision of desk phones and phone credit to aid communication of sputum test results from the laboratory to the clinic staff and from the clinics staff to the patients (enablement) would further reduce the turnaround time for sputum test results. The summary of selected intervention functions that would address the elicited barriers is provided in Table 3.

**Table 3:** Intervention Functions derived from the Behavior Change Wheel targeting elicited barriers to TB treatment initiation

INTERVENTION FUNCTION	HOW THE FUNCTION AFFECTS TARGETED BARRIER	
	Health Facility Barrier	Patient-level Barrier
**Education**	Healthcare workers are made aware about the proportion of patients not initiated on TB treatment at their health facilities and about the outcomes of these patients so that they can institute measures to improve TB treatment initiation.	Patients are educated about TB signs and symptoms as well as benefits of TB treatment to encourage care seeking and reduce stigma.
**Persuasion**	Healthcare workers are encouraged to give adequate patient education so that patients are persuaded to come back to retrieve their sputum test results.	
**Environmental Restructuring**	Healthcare workers deliver sputum samples periodically throughout the day to avoid batched delivery at the end of each clinic day.	Improved TAT-including same-day diagnosis so that patients do not incur additional transport fares to retrieve sputum test results and initiate TB treatment.
	Healthcare workers use improved GeneXpert forms to improve capturing of patient locators.	
	Clinic staff are provided with job aides to enable them to give uniform messages about sputum results retrieval.	Patients are given uniform messages about the TB diagnostic process and sputum results retrieval.
**Enablement**	Clinic and laboratory staff are provided with desk phones and phone credit to enable quick communication of sputum test results.	
**Modeling**		
**Incentivization**	Healthcare workers are given periodic feedback about how well the health facility is performing.	Patients are given cash incentives to encourage them to come back to the health facility to retrieve their sputum test results.
**Restriction**		
**Coercion**		

## Discussion

One of the components of the first pillar of the End TB strategy calls for the early diagnosis and prompt treatment of all persons with TB. Failure to initiate TB treatment after diagnosis is a critical limitation in the provision of TB care that has often been described in high TB burden settings including Uganda [6, 10, 11, 13]. Our descriptive qualitative study sought to understand patient and health system barriers to TB treatment initiation in order to design appropriate interventions.

Our study which was informed by the COM-B model found that barriers to TB treatment initiation existed across all levels of the model (Capability, Opportunity and Motivation). Healthcare workers were not aware that a significant proportion of patients diagnosed with TB at their health facilities were not initiated on TB treatment, which prevented them from instituting interventions to address this problem. On the other hand, there were many missed opportunities to initiate patients on TB treatment which were sometimes a result of patient-level factors, e.g. lack of time and transport fares to retrieve sputum test results, but were often structural e.g. long turnaround times for sputum test results, poor communication of sputum results and poor documentation of patient locators. There were motivational barriers at both the healthcare worker and patient levels. Healthcare workers were disinterested in performing sputum analysis which further increased sputum turnaround time while TB stigma and misconceptions about TB diagnosis made it difficult for patients to accept a TB diagnosis.

Many of our findings have been reported in similar settings. In Uganda, structural barriers like human resource constraints, staff discomfort with handling sputum analysis and inability to follow-up patients who did not return to the health facility after the initial visit all affected healthcare workers’ ability to ensure that patients complete the TB diagnostic cascade [18]. In India and South Africa, documentation of patient particulars at health facilities particularly those using manual reporting tools was inadequate and was associated with low TB treatment initiation [19–21]. In our study, poor recording of patient locators resulted in inability to trace patients not linked to TB treatment even when home visits were attempted. Long turnaround times for sputum results resulting from human resource constraints or weak organizational systems at health facilities have previously been noted as a major cause of pretreatment LFU [18, 22–24]. In Malawi, delays in receiving sputum test results from public health facilities caused patients to resort to alternative care pathways e.g. traditional healers or private practitioners. In our study we also found that coping mechanisms adopted by healthcare workers which included deferring return dates or scheduling of sputum results retrieval to coincide with the next HIV clinic appointment date further delayed treatment initiation. Long turnaround times also interacted with other patient-level factors e.g. lack of transport fares to return to the health facility to decrease TB treatment initiation particularly if patients had to travel more than once to retrieve sputum test results. Shortening sputum test results turnaround times and instituting community-based drug delivery interventions for patients who are not initiated on TB treatment could help alleviate these barriers.

TB stigma hinders care seeking [25–27] treatment initiation [28–31], contact tracing [32] and adherence to TB treatment [25–27]. TB stigma often results in isolation and discrimination of patients with TB. In our study respondents mentioned that they were afraid their family members would not want to live with or share meals with them if they were diagnosed with TB. Evidence-based interventions to reduce TB related stigma e.g. community education and formation of community-based support groups for patients and their families,may contribute to reduction of pre-treatment LFU [33].

Similar to our findings, studies have shown that costs incurred by patients while seeking care for TB are often prohibitive and form a significant barrier to completion of the care cascade [34–36]. In Uganda, a recently concluded study to assess costs incurred by TB patients showed that 53% of households where one of the members had TB experienced catastrophic costs (costs above 20% of their household incomes) while seeking care for TB, the majority of which was spent on transport costs to and from the health facility [37].

A short turnaround time for sputum test results and availability of TB treatment were the main facilitators for TB treatment initiation. Patients who were successfully initiated on TB treatment reported that sputum test results were availed on the day they were evaluated for TB (same-day diagnosis) or on the date of first return. The main value of a short turnaround time may lie in its ability to mitigate several patient-level barriers e.g. transport costs for return visits and time constraints for those who were employed. Once patients received their sputum test results, they were motivated to start TB treatment because the treatment was available and because they believed it would protect their family members from the disease.

Our study used a validated behavior change theory and examined both patients and healthcare workers. We were therefore able to identify additional barriers to TB treatment initiation like batched delivery of sputum samples, inadequate recording of patient locators and lack of effective means of communication between patients and health facilities. We were also able to examine the interaction between patient and health facility level barriers and facilitators in order to understand which barriers may have the greatest impact on our desired behaviour if addressed. Finally, we applied the BCW framework to map possible interventions to address elicited barriers and provide insight into public health interventions that could be implemented by the national TB program. Some of these interventions e.g. the provision of education, improvement of sputum test turnaround times and performance feedback to healthcare workers have been implemented in other settings and have proven useful for improving TB treatment initiation [38–41]. Other incentives like the provision of cash incentives have proved useful for improving retention in care for patients already on TB and/or HIV treatment [42, 43] and should be explored for improvement of treatment initiation among patients diagnosed with TB.

Our study had limitations; we were only able to elicit barriers to treatment initiation among patients who were successfully traced after pre-treatment LFU which could have resulted in selection bias. It is possible that patients who remained untraced experienced even greater barriers which remained undocumented. Future studies should explore the possibility of interviewing family members or close contacts of patients not successfully traced including those who die prior to TB treatment initiation. Further, barriers and facilitators for TB treatment initiation among patients were self-reported and elicited retrospectively. This could have led to recall bias, telescoping and attribution. However, this was minimized by triangulating many data sources (patients, healthcare workers and health managers) and using various methods of data collection (FGDs, KIIs and in-depth interviews). Forthcoming studies should consider enrolling patients prospectively to prevent recall bias and interviewing care givers to strengthen the triangulation of study findings. Third, we did not allow for participant rechecking of transcripts or study findings which could have led to investigator bias in the interpretation of results. However, triangulation of data sources as described above and allowing for a second coder who was part of the study investigators minimized this. Finally, our study did not include patients diagnosed in private health facilities who were likely to experience additional barriers to treatment initiation since the majority of private clinics do not have access to TB treatment and have to refer patients to the public healthcare system. Future studies exploring this phenomenon should include patients diagnosed in the private sector in order to document the barriers and facilitators experienced by this group of patients.

## Conclusion

Our study identified pertinent barriers and facilitators for TB treatment initiation at both patient and health facility level and provides some insight into public health interventions that could be useful for improving TB treatment initiation. The intervention functions identified here should be tested for feasibility.

## Supplementary Information

**Additional file 1.**

## Data Availability

The datasets used and/or analyzed during the current study are available from the corresponding author on reasonable request.

## Abbreviations

COM-B: Capacity, Opportunity, Motivation and Behaviour
FGD: Focus Group Discussion
KII: Key Informant Interviews
HIV: Human Immunodeficiency Syndrome
IDI: In-depth Interview
NTLP: National TB and Leprosy Program
TB: Tuberculosis
WHO: World Health Organization
