# Supplementary material for: Patient and health system level barriers to and facilitators for tuberculosis treatment initiation in Uganda: a qualitative study
Source: BMC Health Serv Res. 2022 Jun 28;22:831. doi: 10.1186/s12913-022-08213-w (PMC9513807; doi:10.1186/s12913-022-08213-w)
Supplement: Supplementary file 1 — Additional file 1. [file 12913_2022_8213_MOESM1_ESM.pdf]

SUPPLEMENTARY FILE 1

IN-DEPTH INTERVIEW GUIDE (FOR PATIENTS)

Study Title: <u>Developing a Theory Informed Intervention to Improve Linkage to Treatment for patients diagnosed with bacteriologically confirmed TB.</u>

<u>Introduction</u>

Dear participant, welcome to this session and thanks for taking the time to talk with us. We are from the Infectious Diseases Institute and will be carrying out a study to understand why some people diagnosed with TB do not start on treatment for the disease. You have been invited to this interview because you are a person who was diagnosed with TB at a public health facility and we believe that your experience with the disease and the processes of care will help us understand why some people are started on TB treatment and others are not. Any information that you give will be strictly confidential so please feel free to share your point of view.

Ethical considerations

- Participation in this study is voluntary and anonymous . No names will be recorded and no individual level information will be shared.
- You can choose to stop participating at any time. We expect not to spend more than 30 minutes during this discussion.
- We will take notes during the discussion and will audio record when possible, to help capture your comments accurately and complete our notes. We will destroy the recordings after we make the notes. We request your permission to audio-record the session.

Introductory Question

1. Please tell us about your background and how you came to be diagnosed with TB

Probes:

- o What signs and symptoms of illness did you present with at the health facility?
- o How long did you have those signs and symptoms before deciding to come to the health facility to seek care?

INTERVIEW QUESTIONS – COM-B FRAMEWORK

CAPACITY

1. Before you came to this health facility, did you know anything about Tuberculosis? ***Probes:***
   - o What causes TB? How does it spread? Who can get infected with TB?
   - o What are its signs and symptoms?
   - o How is TB diagnosed and treated?

2. Before you came to this health facility, did you know which type of health facility to go to get a TB diagnosis or TB treatment?

   Probe:

   - o What type of health facilities offer TB diagnostic and treatment services?

3. How was the process of accessing the results of your sputum test explained to you?

Probes:

- o Did the patient know how long to wait for or when to come back for their result?
- o Did the patient know where to retrieve their sputum result from (which healthcare worker or which clinic) - if they had to come back on a different day?

OPPORTUNITY

1. Did you experience any difficulties coming to the health facility to get tested for TB?
2. Did you experience any difficulties coming back to the health facility to receive the results of your sputum test? If yes, which difficulties?

   Probes:

   - o How far was the health facility from their home (physical accessibility)?
   - o Did the patient have time and resources to go to the health facility?
   - o Was there any influence from family members to be tested or not to be tested for TB?

3. After you left the healthcare worker, did you experience any difficulties in submitting your sputum sample for examination?

   Probes:

   - o Did you know where to submit your sputum sample for examination?
   - o Did you know how to get there (health system navigation)?

4. What amount of time elapsed between the submission of your sputum sample for testing and receiving your sputum results?

   Probes:

   - o Was TAT longer than anticipated? Or shorter than anticipated?
   - o Did patient have to come back more than once to the health facility for their sputum test result?

5. After you received the results of your sputum sample, did you have any difficulties accessing TB treatment from at the health facility?

   Probes:

   - o Did patient know where to go for TB treatment initiation (which healthcare worker or clinic)?

6. What amount of time elapsed between receiving the results of your sputum sample and being started on TB Treatment?

MOTIVATION

1. Do you trust that taking the medications given to you at the health facility will cure you of TB?
2. Do you feel it is important to take all medications as prescribed by the healthcare workers?

FINAL CONCLUDING QUESTION

3. What do you think was most helpful in getting you linked to TB treatment after you were diagnosed with TB? (For patients who were successfully initiated on TB treatment)
4. What do you think could have been done differently to help you get linked to TB treatment after you were diagnosed with TB? (For patients who were not initiated on TB treatment)

FOCUS GROUP DISCUSSION GUIDE (FOR HEALTHCARE WORKERS)

Study Title: <u>Developing a Theory Informed Intervention to Improve Linkage to Treatment for patients diagnosed with bacteriologically confirmed TB.</u>

<u>Introduction</u>

Dear participant, welcome to this session and thanks for taking the time to talk with us. We are from the Infectious Diseases Institute and are carrying out a study to determine how to improve linkage to TB treatment for patients diagnosed with TB. You have been invited to this interview because you are a healthcare worker who is involved in delivery of TB care services at your health facility. Any information that you will be gathered during this focus group discussion will be strictly confidential so please feel free to share your point of view. In order to maintain confidentiality during the interview, we shall refer to each other by our initials and not by our names. We will take notes during the interview and will also record it using a tape recorder.

GROUND RULES

- We expect to take not more than one hour for this discussion.
- The most important rule is that only one person speaks at a time. There may be a temptation to jump in when someone is talking but please wait until they have finished.
- You do not have to speak in any particular order. When you do have something to say, please do so. There are a number of you in the group and it is important that we obtain the views of each of you.
- There are no right or wrong answers. You do not have to agree with the views of other people in the group.
- We will start with introducing ourselves to each other.

INTRODUCTION

CAPACITY

1. I am just going to give you a couple of minutes to think about your experience of giving care for TB at this health facility. From this experience, how do you think linkage to TB treatment for patients diagnosed with TB is doing?

Probe:

- o Ask healthcare workers to qualify their yes or no answers by contrasting NTLP guidelines on TB treatment initiation with data from their own TB clinic.
- o Ask about mechanisms present at the health facility to track linkage to treatment among patients diagnosed with TB.

OPPORTUNITY

2. Take a moment and think about the different aspects of workflow at your health facility. What aspects of workflow determine whether or not patients diagnosed with TB are linked to TB treatment?

   Probe

   - Ask about all aspects of TB care e.g. screening, sample collection, sample testing, results communication.

3. What patient follow-up mechanisms do you have in place at this health facility to ensure that all patients diagnosed with TB are linked to TB treatment?

MOTIVATION:

1. Do you think that it is your responsibility of the healthcare worker to initiate all patients diagnosed with TB on TB treatment? If yes, why? If not, whynot?

FINAL QUESTION

Of all the things we've discussed today, what would you say is the most important issue that should be addressed in order to improve linkage to TB treatment for patients diagnosed with TB?

- Note: First explain to the group the intervention functions of the COM-B model e.g., environmental restructuring, modelling, enablement, coercion, incentivisation and education.

CONCLUSION

Thank you for participating. This has been a very successful discussion and we hope that you have found it interesting. I would like to remind you that any comments featuring in this report will be anonymous

KEY INFORMANT INTERVIEW GUIDE (FOR HEALTHCARE MANAGERS)

Study Title: <u>Developing a Theory Informed Intervention to Improve Linkage to Treatment for patients diagnosed with bacteriologically confirmed TB.</u>

<u>Introduction</u>

Dear participant, welcome to this session and thanks for taking the time to talk with us. We are from the Infectious Diseases Institute and are carrying out a study to determine how to improve linkage to TB treatment for patients diagnosed with TB. You have been invited to this interview because you are a healthcare manager who works in area that delivers TB care services e.g. laboratory, outpatient clinic or HIV clinic. We believe that your knowledge of and experience with the processes of care will help us understand why some people are successfully initiated on TB treatment and others are not. Any information that will be gathered during this focus group discussion will be strictly confidential so please feel free to share your point of view. In order to maintain confidentiality during the interview, we shall refer to you by our initials and not by your name. We will take notes during the interview and will also record it using a tape recorder.

Ethical considerations

- Participation in this study is voluntary and anonymous. No names will be recorded and no individual level information will be shared.
- You can choose to stop participating at any time. We expect not to spend more than 30 minutes during this discussion.
- We will take notes during the discussion and will audio record when possible, to help capture your comments accurately and complete our notes. We will destroy the recordings after we make the notes. We request your permission to audio-record the session.

INTERVIEW QUESTIONS – COM-B FRAMEWORK

Introductory Question

1. How do you think your health facility is performing as regards to linkage to TB treatment for patients diagnosed with TB?

Probe:

- Ask about monthly health facility performance on linkage to TB treatment for the past three months.
- Ask about mechanisms present at the health facility to track linkage to treatment among patients diagnosed with TB.

CAPACITY

1. At your health facility, how soon after a bacteriological confirmation of TB should patients be initiated on TB treatment?

Probe:

- Ask about for knowledge of NTLP guidelines on how soon a patient who is diagnosed with TB can be started on treatment.

2. What aspects of the health facility workflow influence whether or not patients diagnosed with TB are linked to TB treatment?

OPPORTUNITY

1. What patient follow-up mechanisms do you have in place to ensure that patients are linked to TB treatment?

Probe:

- Ask about for immediate mechanisms (results TAT, results sharing with patients) and long-term mechanisms e.g. community-based follow up.

2. What monitoring systems do you have in place to track what proportion of patients with bacteriologically confirmed TB have been started on TB treatment?

Probe:

- Ask about any manual or electronic mechanisms to reconcile TB lab registers to TB treatment registers on a periodic basis at the health facility
- Ask about inclusion of data on linkage to TB treatment in periodic reports to the NTLP e.g. weekly, monthly or quarterly reports.

MOTIVATION

1. Do you think that it is the responsibility of the healthcare worker to ensure that patients diagnosed with TB are initiated on TB treatment?

Probe:

- Probe for perceived beliefs about the responsibility for the processes involved in ensuring that patients are started on TB treatment.

2. Do you think that it is possible to initiate all patients diagnosed with TB on treatment? If not, why not? If yes, what are the benefits of initiating patients on TB treatment as soon as they are diagnosed with TB?

Probe:

- Probe for beliefs about the benefits of initiating patients diagnosed with TB on treatment for the patients, their family and the wider community.

CONCLUSION

We will now go through the COM-B model so that we are familiar with the intervention components e.g., environmental restructuring, modelling, enablement, coercion, incentivisation and education.

1. Which intervention components from the COM-B model could best overcome the modifiable barriers and enhance enablers for linkage to TB treatment at your health facility?
